# Gait analysis in cerebral palsy (2005–2025): a bibliometric mapping of research trends, collaboration networks, and emerging technologies

**DOI:** 10.3389/fneur.2026.1899328

**Published:** 2026-07-07

**Authors:** Zhe Wang, Xiao Liu

**Affiliations:** 1 Department of Pediatric Surgery, Qilu Hospital of Shandong University, Jinan, China; 2 Department of Neuro Surgery, Qilu Hospital of Shandong University, Jinan, China

**Keywords:** bibliometric analysis, cerebral palsy, crouch gait, gait analysis, research trends, wearable sensors

## Abstract

**Background:**

Gait dysfunction is a hallmark of cerebral palsy (CP) limiting mobility and quality of life. Despite growing research, a systematic synthesis of the scientific landscape of CP gait analysis is lacking.

**Methods:**

We systematically searched the Web of Science Core Collection (2005–2025) following PRISMA guidelines. VOSviewer and CiteSpace were used for co-authorship, co-occurrence, clustering, burst detection, and dual-map overlay analyses. To verify the robustness of our findings, a secondary validation analysis was performed using Scopus (Elsevier) with an equivalent search strategy.

**Results:**

1,404 publications were identified; annual output grew from 26 to 112. The USA was most productive (376 papers, h-index 45). Gait & Posture published the most articles (207). Keyword clustering identified 10 clusters, with “crouch gait,” “reliability,” and “gross motor function” as persistent themes. Recent bursts include “wearable sensors,” “musculoskeletal modeling,” and “artificial intelligence.” Notably, intervention studies linking gait analysis to patient-centered outcomes remain underrepresented.

**Conclusion:**

This bibliometric analysis reveals a maturing field with growing research interest in technology-assisted rehabilitation, as suggested by recent keyword bursts. Emerging technologies (wearables, AI, musculoskeletal modeling) warrant prioritization in future clinical research, although their current adoption remains limited. The persistent evidence gap between gait measurement and clinical decision-making calls for multi-center validation and real-world implementation studies.

**Systematic review registration:**

Open Science Framework (OSF) (DOI: 10.17605/OSF.IO/CT27P).

## 1 Introduction

Cerebral palsy (CP) is one of the most common neurodevelopmental disorders in children, with a global prevalence of approximately 2 per 1,000 live births (1, 2). Gait impairment is one of the most disabling consequences of CP, directly restricting independence in daily activities, limiting social participation, and diminishing health-related quality of life (3, 4). As a result, quantitative gait analysis has become a longstanding core research theme in CP. Over the past two decades, CP gait research has shifted from qualitative clinical observation to three-dimensional motion capture, from surface electromyography to wearable sensors, and from single-parameter assessment to multi-modal integrated analysis (5, 6). These technologies enable the objective, quantitative assessment of lower-limb joint kinematics, kinetics, muscle activation patterns, and energy expenditure during gait, thereby informing and refining clinical decision-making (7).

Given this technological expansion, the volume of CP gait literature has grown substantially. For example, as will be shown in our results, annual publications increased from 26 in 2005 to 112 in 2025—a compound annual growth rate of 8.6%. Despite this rapid growth, a systematic mapping of the overall scientific landscape, including collaboration structures, thematic evolution, and emerging directions, has not yet been performed specifically for this subfield.

Bibliometric analysis uses quantitative methods to interrogate large-scale bibliographic datasets, tracking research evolution, collaborative structures, and leading contributors (8). Specialized software tools such as CiteSpace and VOSviewer are commonly employed to perform science mapping and network visualization (9–11). By drawing on standardized bibliographic data, bibliometric analyses provide reproducible, macro-level perspectives that complement traditional narrative reviews (12).

Several bibliometric studies have investigated CP research broadly. Hu et al. analysed CP publications from 2003 to 2022 and identified “gait” as the most frequent keyword, yet they did not isolate gait analysis as a distinct domain (13). Jiang et al. examined diagnosis and therapies without differentiating gait-specific literature (14). Most recently, Qian et al. focused on non-pharmacological gait rehabilitation interventions across neurodevelopmental disorders, without specifically targeting CP or instrumented methods (15). To our knowledge, the present study is the first bibliometric analysis specifically centered on IGA in CP, integrating knowledge structure mapping, collaboration network analysis, and emerging trend detection within a unified framework. Unlike traditional narrative reviews, bibliometric analysis allows for unbiased, reproducible, and large-scale synthesis of publication patterns, collaboration networks, and topic evolution, offering a macro-level perspective that complements existing qualitative reviews of CP gait research.

In this study, we used VOSviewer and CiteSpace to conduct a multidimensional bibliometric analysis of CP gait research. Specifically, we aim to: (1) characterize publication output and temporal trends; (2) identify core countries, institutions, authors, and journals; (3) map collaboration networks; and (4) discern dominant research hotspots and emerging directions. By combining quantitative mapping with qualitative interpretation, this study aims to provide clinicians, researchers, and funders with an evidence-based reference for future investments in CP gait research.

## 2 Materials and methods

### 2.1 Data source and search strategy

Data were retrieved from the Web of Science Core Collection (WoSCC) on 21 May 2026. The following search syntax was applied: TS = (“cerebral palsy” OR “CP”) AND TS = (“gait” OR “walking” OR “gait analysis”). The publication period was set from January 2005 to December 2025, covering two decades of substantial growth in CP gait research. Language was restricted to English. This systematic review was conducted and reported in accordance with the Preferred Reporting Items for Systematic Reviews and Meta-Analyses (PRISMA) 2020 statement (16). The PRISMA checklist is provided as Supplementary Table S1. No ethical approval was required because all data were derived from published literature. This systematic review protocol was preregistered on the Open Science Framework (OSF) (DOI: 10.17605/OSF.IO/CT27P).

### 2.2 Inclusion criteria and data cleaning

Eligible records met the following criteria: (1) the study focused on both CP and gait analysis; (2) the document type was an original article or review (meeting abstracts, letters, and editorials were excluded). Titles and abstracts of the retrieved records were screened independently by two authors to confirm eligibility; any disagreements were resolved through discussion. Data were exported as “full record and cited references” from the database. After export, duplicate records were identified and removed. After deduplication, two rounds of data normalization were performed. First, author names, institutional affiliations, and keywords were manually harmonized using a dictionary file in VOSviewer (e.g., “CP” to “cerebral palsy”; “3D gait analysis” to “three-dimensional gait analysis”). Second, CiteSpace’s built-in alias function was used to merge variant forms of the same entity. The PRISMA framework was applied solely to the literature search and record-selection process; no qualitative risk-of-bias assessment of individual studies was performed, as this is beyond the scope of a bibliometric analysis.

### 2.3 Analysis tools and parameters

CiteSpace (version 7.0 R0; Chen, 2006) was used for collaboration network analysis, journal co-citation analysis, dual-map overlay, keyword co-occurrence and clustering, burst detection, and timeline visualization (10). The network was pruned using the Pathfinder algorithm to reduce visual clutter while preserving backbone structure (17). Keyword clusters were labelled using the log-likelihood ratio (LLR) algorithm (18). Burst detection identified keywords with sudden frequency increases (minimum duration: 1 year). VOSviewer (version 1.6.20) was employed to complement and validate the CiteSpace networks, using fractional counting and association strength normalization for co-authorship and co-occurrence analyses (11).

Detailed parameter settings (including g-index, k-core values, and iterative calibration procedures) are provided in Supplementary material. The combined use of both tools allows cross-validation of network structures and enhances the robustness of the mapping. VOSviewer was chosen for its strength in network visualization and fractional counting, while CiteSpace was employed for its advanced burst detection and dual-map overlay capabilities.

### 2.4 Scopus validation

To assess the robustness of our bibliometric findings and to identify potential database-specific biases, we performed a secondary validation analysis using Scopus (Elsevier). The equivalent search strategy applied in Scopus was: TITLE-ABS-KEY (“cerebral palsy” OR “cp”) AND TITLE-ABS-KEY (gait OR walking OR “gait analysis”). The same time frame (2005–2025), document types (articles and reviews), and language restriction (English) were applied. Data were exported in CSV format and cleaned using identical normalization protocols (DOI-based matching, author name harmonization, and institution merging). Key indicators—including publication trends, core journals, leading countries and institutions, keyword clusters, citation bursts, and highly cited papers—were compared between WoSCC and Scopus. Overlap between the two databases was quantified at the article level using DOI as the primary matching key, with title–first-author–year fuzzy matching applied for records lacking a DOI. Divergences were systematically recorded and interpreted in the context of each database’s coverage focus.

### 2.5 Analytical approach

Annual publication trends were analyzed using linear regression in R (version 4.3.0, R Core Team, 2023), with the Durbin-Watson statistic calculated to test for autocorrelation in the residuals. In the collaboration networks, node size reflected the number of publications (or citation count), while link thickness indicated the strength of collaborative ties. Temporal evolution was visualized through timeline views of keyword clusters, and keyword burst detection was applied to identify terms with a sudden increase in frequency. The significance level for regression coefficients was set at *p* < 0.05.

## 3 Results

### 3.1 General publication output, citation trends, and future projections

**Figure 1:**
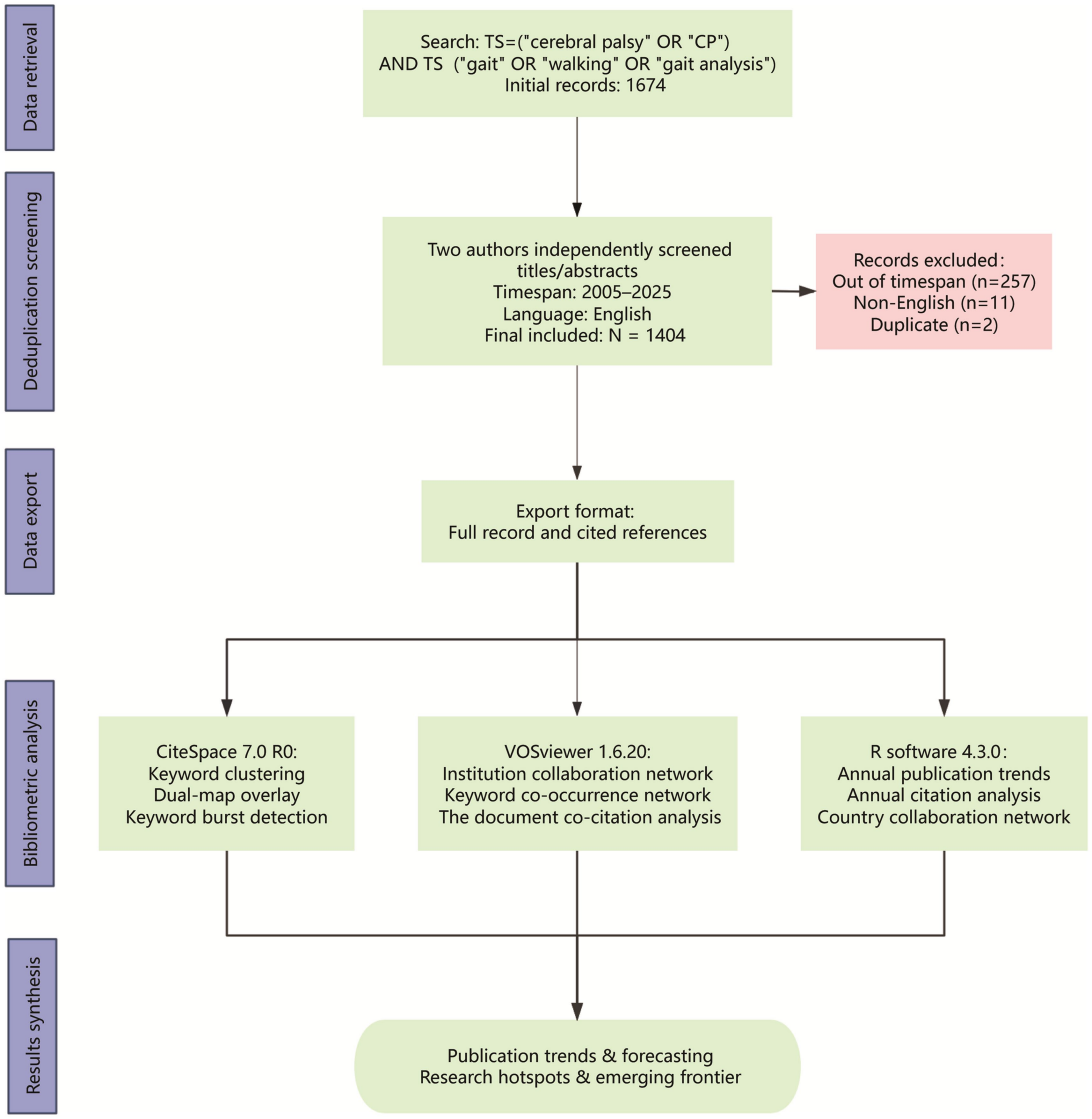
PRISMA 2020 flow diagram showing the identification, screening, and inclusion process.

**Figure 2:**
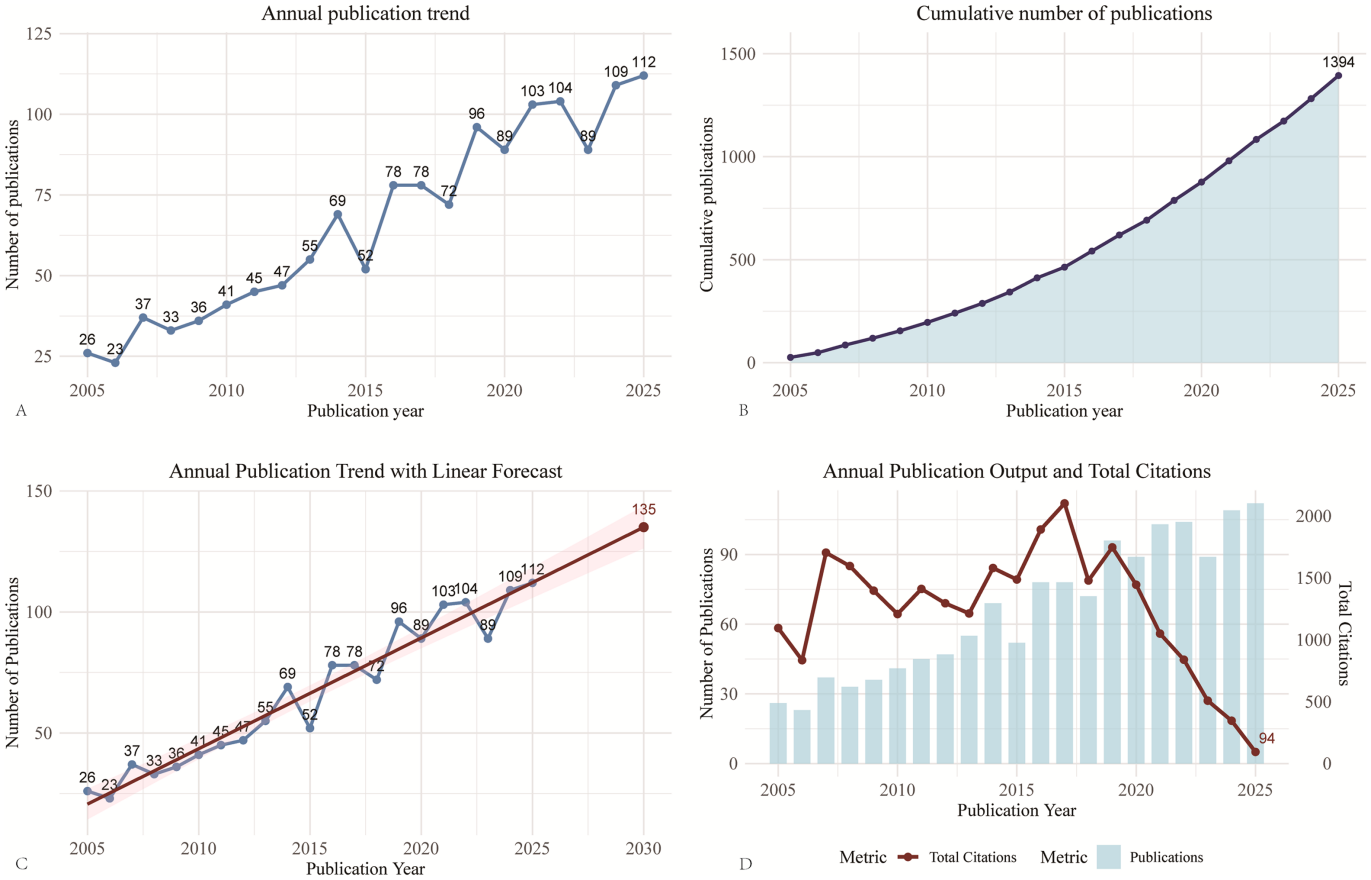
Annual publication output and citation trends. **(A)** Number of articles published per year. **(B)** Linear regression analysis showing a significant upward trend (*R*^2^ = 0.9432, *p* < 0.001). **(C)** Forecasted annual publications through 2030 based on the linear model. **(D)** Total citations aggregated by publication year.

A total of 1,404 articles published between January 2005 and December 2025 were included. The PRISMA flow diagram detailing the screening and selection process is shown in Figure 1. The annual publication count increased steadily from 26 in 2005 to 112 in 2025, corresponding to a compound annual growth rate of 8.6% (Figure 2A). Linear regression analysis revealed a significant upward trend for publications (*R*^2^ = 0.9432, *p* < 0.001) (Figure 2B). Based on the linear regression model, the forecasted number of annual publications for 2030 was 135 (Figure 2C). These projections should be interpreted with caution, as they are based solely on historical linear trends. Figure 2D illustrates the distribution of total citations aggregated by publication year, reflecting the cumulative scholarly impact of papers published in each year.

### 3.2 Core countries, institutions, authors, and journals

The United States was the most productive country (376 publications, 26.8%), followed by the Netherlands (108, 7.7%). In terms of citation impact, the United States also ranked first in total citations (7,969) and H-index (45), while Australia achieved the highest citations per article (30.03). The G-index, which gives more weight to highly cited publications, was also calculated for journals to complement the H-index. Detailed publication and citation metrics for the top 10 most productive countries are presented in Table 1. These data reveal a dual pattern: while the USA dominates in absolute output and total citations, several European countries (Netherlands, Belgium, Switzerland) achieve higher citations per article, suggesting a “small country, high impact” profile. The concentration of top performing countries in Western Europe and North America indicates regional clustering of research capacity.

**Table 1:** Top 10 most productive countries in CP gait analysis research.

Rank	Countries	Record count	No. of times cited	No. of times cited (per article)	H-index
1	USA	376	7,969	21.19	45
2	Netherlands	108	2,818	26.09	35
3	Switzerland	84	1,602	19.07	23
4	Italy	78	1,607	20.6	23
5	Belgium	74	1886	25.49	28
6	Germany	71	1,407	19.82	21
7	France	68	1,036	15.24	17
8	South Korea	68	1,270	18.68	20
9	Australia	65	1952	30.03	26
10	Canada	65	1,342	20.65	22

At the institutional level, the most productive institution was Vrije Universiteit Amsterdam (Netherlands, 77 publications), followed by KU Leuven (Belgium, 55). In terms of citation impact, Vrije Universiteit Amsterdam also ranked first in total citations (2,104) and H-index (30), while Gillette Children‘s Specialty Healthcare achieved the highest citations per article (32.48). Detailed publication and citation metrics for the top 10 most productive institutions are presented in Table 2. Notably, Vrije Universiteit Amsterdam and KU Leuven are not only the top two producers but also central nodes in the institutional collaboration network (see Supplementary Figure S1). Their strong co-authorship ties with University Hospital Leuven (318 links) suggest a tightly integrated regional hub. Gillette Children’s Specialty Healthcare, despite ranking seventh in output, leads in citations per article (32.48), indicating high per-study influence.

**Table 2:** Top 10 most productive institutions in CP gait analysis research.

Rank	Institutions	Country	Record count	No. of times cited	No. of times cited (per article)	H-index
1	Vrije Universiteit Amsterdam	Netherlands	77	2,104	27.32	30
2	KU Leuven	Belgium	55	1,431	26.02	24
3	University Hospital Leuven	Belgium	42	1,203	28.64	21
4	Egyptian Knowledge Bank (EKB)	Egypt	39	524	13.44	15
5	University of Amsterdam	Netherlands	39	657	16.85	16
6	Cairo University	Egypt	36	502	13.94	15
7	Gillette Children’s Specialty Healthcare	USA	29	942	32.48	14
8	Ruprecht Karls University Heidelberg	Germany	29	380	13.1	12
9	Central Remedial Clinic	Ireland	28	392	14	12
10	University of Minnesota System	USA	28	843	30.11	13

Among individual authors, Desloovere K (KU Leuven, Belgium) was the most productive (46 publications), followed by Buizer AI (Vrije Universiteit Amsterdam, 28). In terms of citation impact, Desloovere K had the highest total citations (1,264) and H-index (19), while Schwartz MH achieved the highest citations per article (41.27). Detailed publication and citation metrics for the top 10 most productive authors are presented in Table 3. Desloovere K and Harlaar J exhibit the highest citation impact among authors, reflecting their foundational contributions. Schwartz MH, with the highest citations per article (41.27), demonstrates that a smaller body of highly influential work can achieve comparable overall recognition.

**Table 3:** Top 10 most productive authors in CP gait analysis research.

Rank	Authors	Institutions	Record count	No. of times cited	No. of times cited (per article)	H-index
1	Desloovere K	KU Leuven	46	1,264	27.48	22
2	Buizer AI	Vrije Universiteit Amsterdam	28	588	21	13
3	Lerner ZF	Northern Arizona University	28	913	32.61	15
4	Van Der Krogt MM	Vrije Universiteit Amsterdam	27	721	26.7	16
5	Harlaar J	Vrije Universiteit Amsterdam	26	932	35.85	19
6	Molenaers G	KU Leuven	25	826	33.04	17
7	Armand S	University of Geneva	23	545	23.7	12
8	Miller F	Nemours Alfred I. DuPont Hospital for Children	23	464	20.17	11
9	Galli M	Polytechnic University of Milan	22	523	23.77	13
10	Schwartz MH	Gillette Children’s Specialty Healthcare	22	908	41.27	14

Regarding journals, *Gait & Posture* was the most productive outlet (207 articles), followed by *Developmental Medicine and Child Neurology* (81). In terms of citation impact, *Gait & Posture* also ranked first in total citations (5,119) and H-index (41), while *Developmental Medicine and Child Neurology* had the highest average citations per article (32.64) and the highest impact factor (4.3, JCR Q1 in Pediatrics). Detailed publication and citation metrics for the top 10 most productive journals are presented in Table 4. *Gait & Posture* serves as the discipline’s core specialized outlet, while *Developmental Medicine and Child Neurology* achieves both the highest impact factor and the highest citations per article, underscoring the translational value of high-impact pediatric journals.

**Table 4:** Top 10 most productive journals in CP gait analysis research.

Rank	Journals	Record count	Number of time cited	Average per item	H-index	G-index	IF(2025)	JCR
1	*Gait & Posture*	207	5,119	24.73	41	42	2.4	CLINICAL NEUROLOGY Q3; ENGINEERING BIOMEDICAL Q3; SPORT SCIENCES Q2
2	*Developmental Medicine & Child Neurology*	81	2,644	32.64	30	31	4.3	CLINICAL NEUROLOGY Q2; PEDIATRICS Q1
3	*Clinical Biomechanics*	49	677	13.82	17	17	1.4	ENGINEERING BIOMEDICAL Q4; SPORT SCIENCES Q4
4	*Pediatric Physical Therapy*	48	383	7.98	14	17	1.5	REHABILITATION Q3
5	*PLOS ONE*	38	670	17.63	15	18	2.6	MULTIDISCIPLINARY SCIENCES Q2
6	*Journal of Pediatric Orthopaedics*	34	1,069	31.44	16	17	1.5	ORTHOPEDICS Q3; PEDIATRICS Q2
7	*Research in Developmental Disabilities*	30	813	27.1	18	20	2.6	EDUCATION SPECIAL Q2; REHABILITATION Q2
8	*Developmental Neurorehabilitation*	26	356	13.69	10	15	1.7	CLINICAL NEUROLOGY Q3; PEDIATRICS Q3; REHABILITATION Q3
9	*Disability and Rehabilitation*	26	650	25	13	22	2	REHABILITATION Q2; SPORT SCIENCES Q2
10	*NeuroRehabilitation*	25	348	13.92	12	17	1.8	CLINICAL NEUROLOGY Q3; REHABILITATION Q2

Between 2005 and 2025, the 10 most frequently cited articles in the field of CP gait analysis accumulated a total of 2,463 citations, with a mean of 246.3 citations per paper (Table 5). The highest-cited work was by Wren et al. in the *Journal of Pediatric Orthopaedics*, which reported the prevalence of specific gait abnormalities in children with CP (400 citations) (20). The second and third positions were occupied by Opheim et al. (21) on walking function, pain, and fatigue in adults with CP (304 citations) and by Steele et al. (22) on muscle synergies and neuromuscular control during gait (313 citations), both published in *Developmental Medicine & Child Neurology*. In total, this journal accounted for four of the top-10 papers, accumulating 993 citations, underscoring its leading role in the domain. The remaining high-impact papers covered topics such as the relationship between spasticity, strength, and gait (19), clinical gait analysis methods (23), reliability of walking tests (24, 25), systematic reviews on gait decline and intervention effectiveness (4), and partial body weight supported treadmill training (26). Notably, review articles (*n* = 3) contributed an average of 206 citations each, reflecting the sustained demand for synthesized evidence in this area. Chronologically, six papers were published between 2005 and 2009, and four between 2014 and 2016, indicating that earlier foundational studies continue to exert substantial academic influence.

**Table 5:** Top 10 most cited articles in CP gait analysis research.

Rank	Article title	Times cited	Authors	Journals	Publication year	DOI
1	Prevalence of specific gait abnormalities in children with cerebral palsy - Influence of cerebral palsy subtype, age, and previous surgery	400	Wren, T	*Journal of Pediatric Orthopaedics*	2005	10.1097/00004694-200501000-00018
2	Walking function, pain, and fatigue in adults with cerebral palsy: a 7-year follow-up study	304	Opheim, A	*Developmental Medicine and Child Neurology*	2009	10.1111/j.1469-8749.2008.03250.x
3	Muscle synergies and complexity of neuromuscular control during gait in cerebral palsy	313	Steele, KM	*Developmental Medicine and Child Neurology*	2015	10.1111/dmcn.12826
4	Relationships between spasticity, strength, gait, and the GMFM-66 in persons with spastic diplegia cerebral palsy	262	Ross, SA	*Archives of Physical Medicine and Rehabilitation*	2007	10.1016/j.apmr.2007.06.011
5	Gait analysis in children with cerebral palsy	246	Armand, S	*EFORT Open Reviews*	2016	10.1302/2058-5241.1.000052
6	Test–retest reliability of the 10-metre fast walk test and 6-min walk test in ambulatory school-aged children with cerebral palsy	223	Thompson, P	*Developmental Medicine and Child Neurology*	2008	10.1111/j.1469-8749.2008.02048.x
7	Gait function and decline in adults with cerebral palsy: a systematic review	179	Morgan, P	*Disability and Rehabilitation*	2014	10.3109/09638288.2013.775359
8	The six-minute walk test for children with cerebral palsy	180	Maher, CA	*International Journal of Rehabilitation Research*	2008	10.1097/MRR.0b013e32830150f9
9	Effectiveness of Rehabilitation Interventions to Improve Gait Speed in Children With Cerebral Palsy: Systematic Review and Meta-analysis	194	Moreau, NG	*Physical Therapy*	2016	10.2522/ptj.20150401
10	Partial body-weight-supported treadmill training can improve walking in children with cerebral palsy: a clinical controlled trial	162	Dodd, KJ	*Developmental Medicine and Child Neurology*	2007	10.1111/j.1469-8749.2007.00101.x

### 3.3 Collaboration network analysis

**Figure 3:**
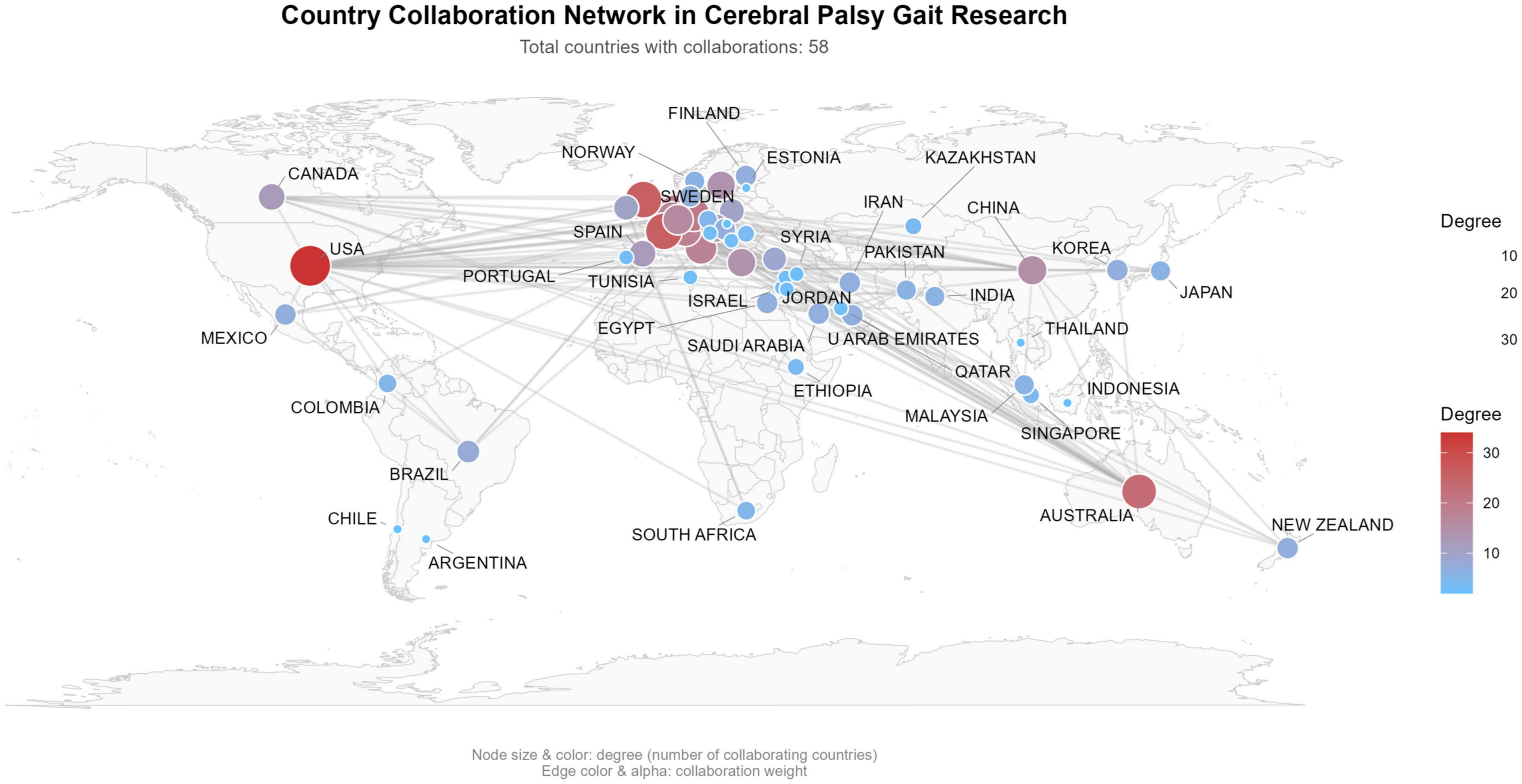
Country collaboration network. Nodes represent countries; node size reflects publication volume; link thickness indicates collaboration strength.

The country collaboration network comprised 58 nodes and 254 edges. The strongest collaborative ties were observed between the USA and Canada (8 links), followed by the USA–United Kingdom (5 links) and USA–Australia (3 links), as shown in Figure 3. The institution collaboration network (see Supplementary Figure S1) comprises 975 nodes and 3,318 edges, with the strongest tie between KU Leuven and University Hospital Leuven (318 links), reinforcing the dominance of the Belgian–Dutch collaboration axis.

### 3.4 Keyword co-occurrence and clustering analysis

**Figure 4:**
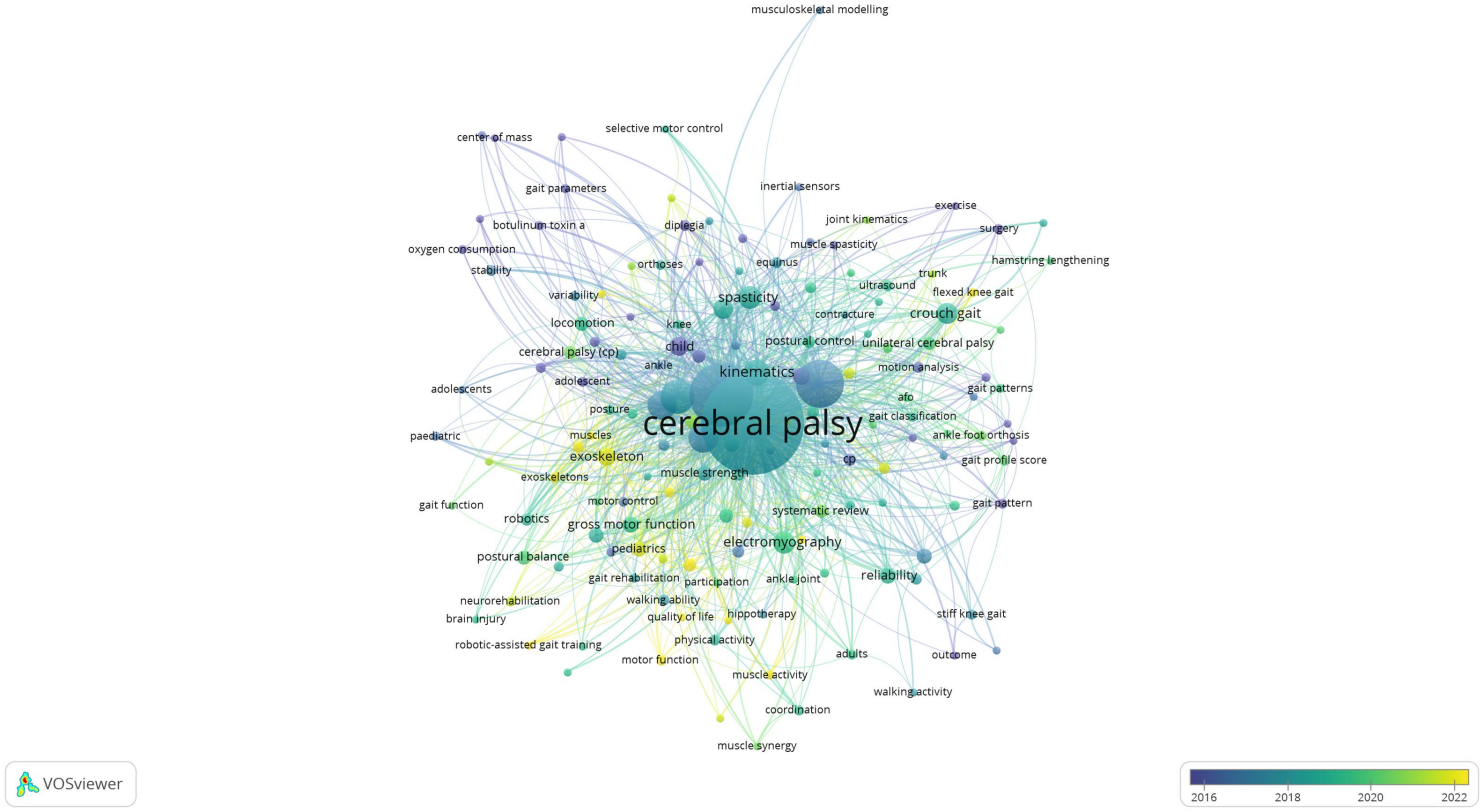
Keyword co-occurrence network. Nodes are keywords that appeared together in at least 5 articles; node size reflects frequency; edge thickness indicates co-occurrence strength. The network contains 486 nodes and 3,670 edges.

The keyword co-occurrence network included 486 nodes and 3,670 edges (Figure 4). The most frequent keywords—"cerebral palsy” (825), “children” (331), “reliability” (282), and “gross motor function” (224)—indicate that methodological dependability and functional outcomes are central concerns. Network modularity (*Q* = 0.3294) and mean silhouette (*S* = 0.5046) indicate well-defined cluster structures with moderate cross-topic overlap.

**Figure 5:**
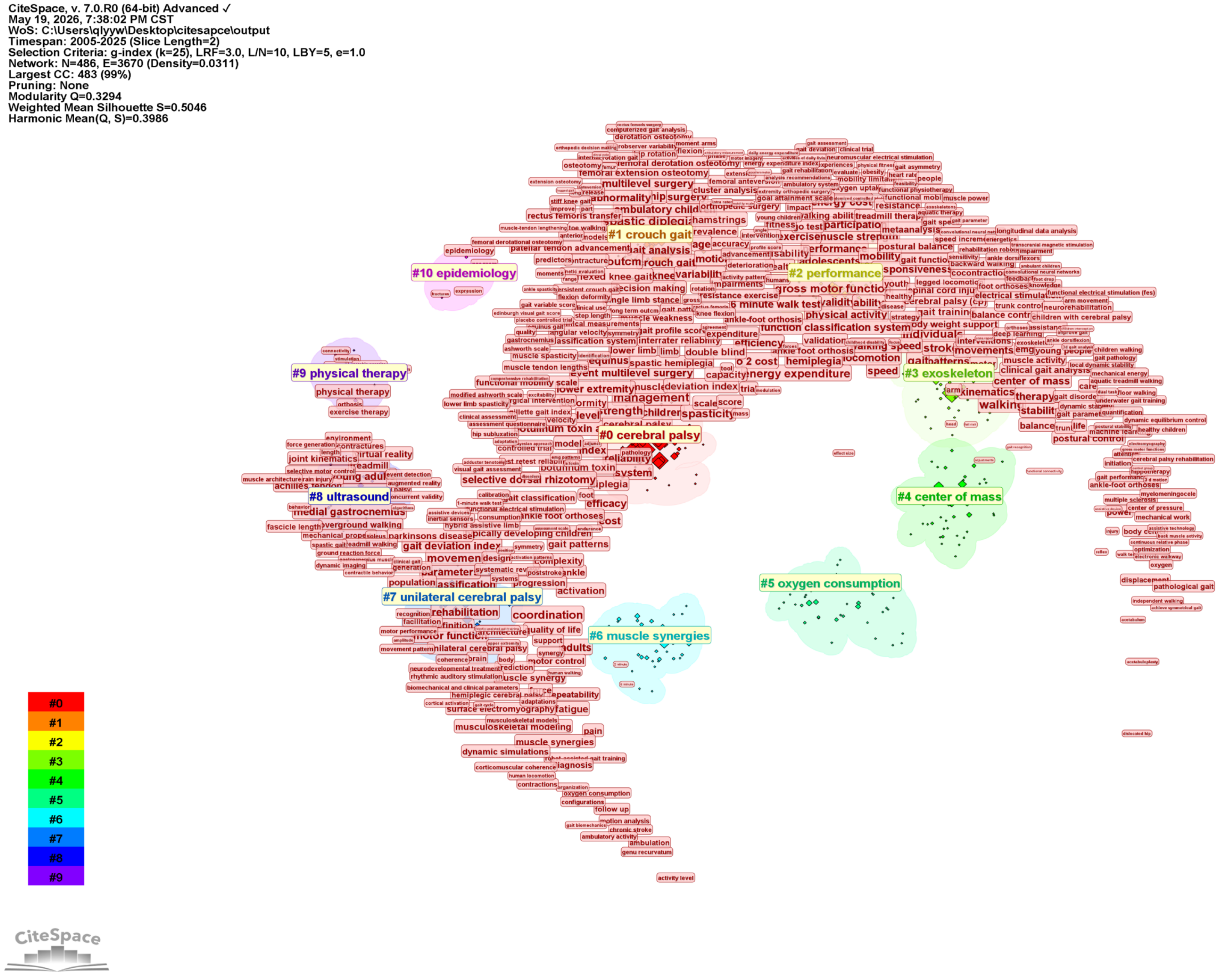
Keyword clustering analysis using the log-likelihood ratio (LLR) algorithm. Ten major clusters are color-coded, with cluster #0 (“cerebral palsy”) and #1 (“crouch gait”) being the largest. Network modularity *Q* = 0.3294, mean silhouette *S* = 0.5046.

Keyword clustering (LLR algorithm) generated 10 major clusters (Figure 5). The largest cluster (#0 “cerebral palsy” size = 75) comprised terms such as “reliability” and “children” suggesting that early research focused on measurement reproducibility and general population description. Cluster #1 (“crouch gait” size = 73) centered on “kinematics” and “spastic diplegia” reflecting the longstanding biomechanical emphasis. Notably clusters related to “exoskeleton” and “center of mass” emerged after 2015 indicating a shift toward intervention-based and postural control research. The temporal continuity of clusters #0 and #6 (“gait analysis”) confirms that these themes form the persistent knowledge backbone of the field.

### 3.5 Keyword burst detection

**Figure 6:**
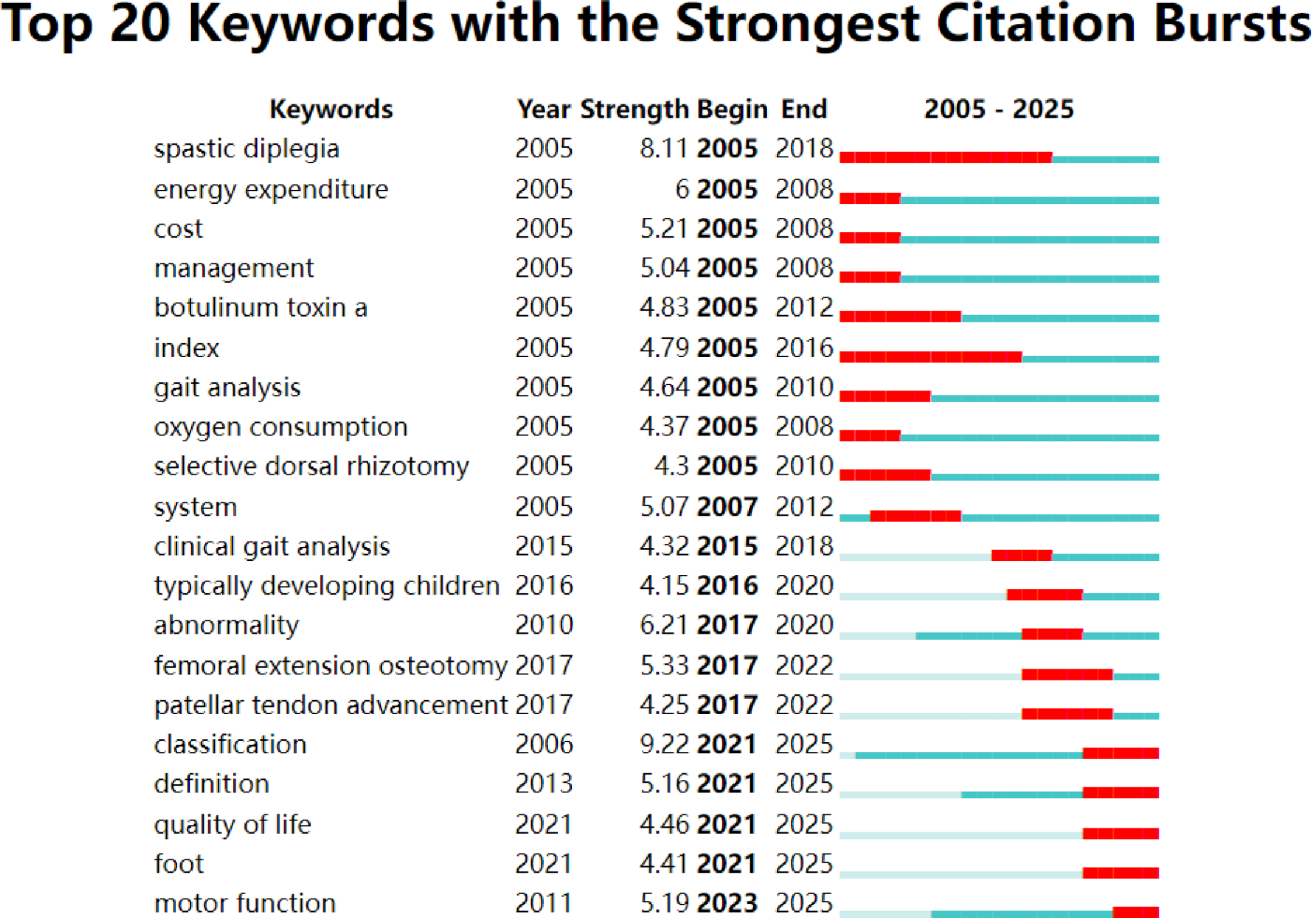
Top 20 keywords with the strongest citation bursts. Blue bars indicate the time period, and red bars mark the burst duration.

Burst detection identified 20 keywords with significant citation surges (Figure 6). The strongest burst was observed for “classification” (strength = 9.22, burst period: 2021–2025), followed by “spastic” (strength = 8.11, burst period: 2005–2018). Recent bursts (2022–2025) included “motor function” (strength = 5.19), “definition” (strength = 5.16), and “quality of life” (strength = 4.46), reflecting emerging research frontiers. Importantly, the appearance of “wearable sensors,” “artificial intelligence,” and “musculoskeletal modeling” as burst terms indicates growing research interest rather than established clinical adoption. These technologies remain predominantly in proof-of-concept and small-scale validation phases, as suggested by their relatively low overall frequencies.

### 3.6 Co-citation and knowledge base analysis

**Figure 7:**
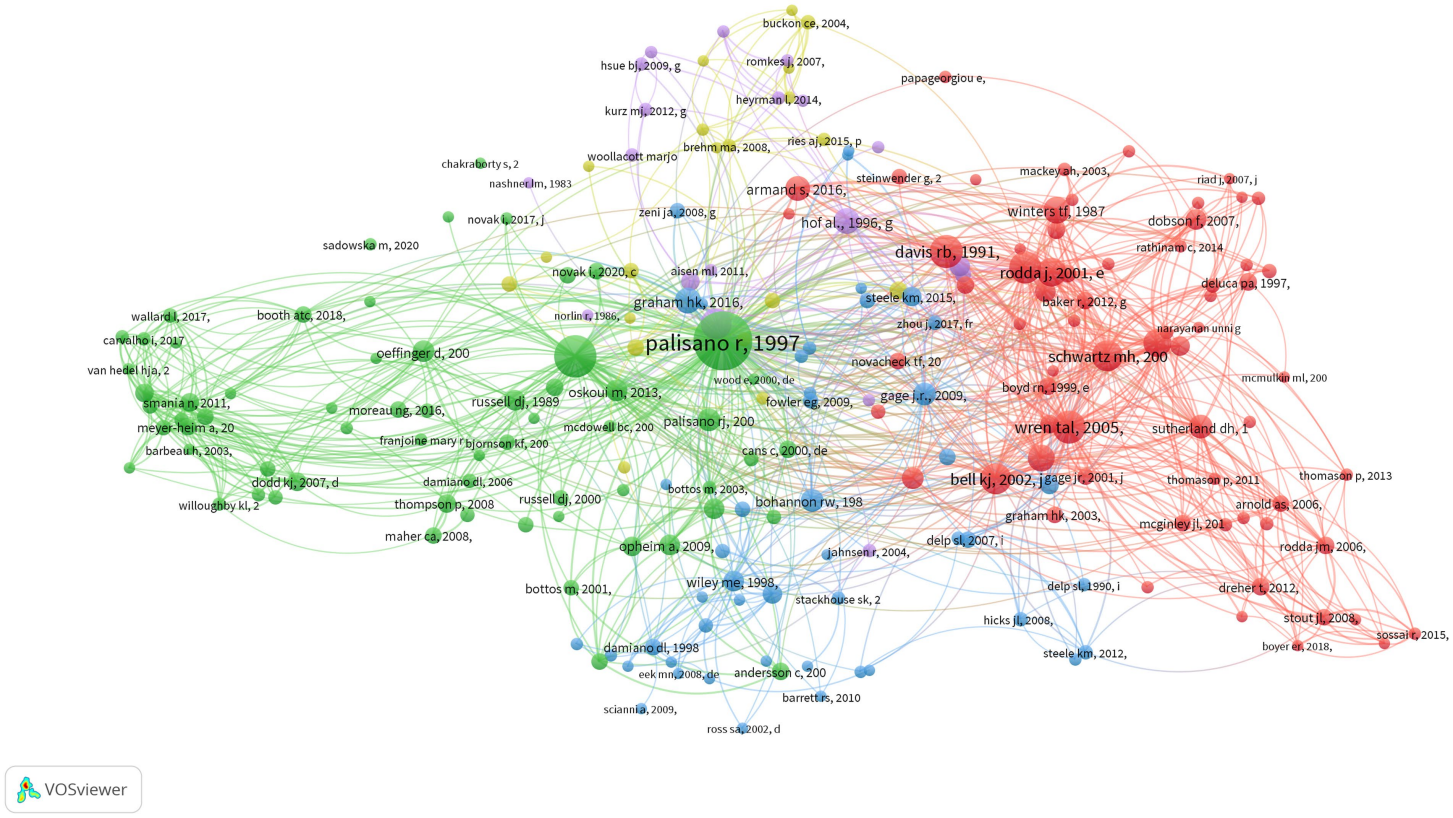
Document co-citation network. Co-cited references form the intellectual base of the field.

The document co-citation analysis generated a network of 217 cited references (Figure 7). The most highly co-cited reference was palisano r, 1997, dev med child neurol, v39, p214 (co-citation count = 337), followed by rosenbaum p, 2007, dev med child neurol, v49, p8 (co-citation count = 192). Clustering of co-cited references identified foundational knowledge domains, including gait biomechanics and clinical gait analysis and functional assessment and quality of life.

### 3.7 Funding agencies

The United States Department of Health and Human Services funded 92 papers, followed by the National Institutes of Health (NIH, 87 papers), details shown in Table 6. Within the NIH, the Eunice Kennedy Shriver National Institute of Child Health and Human Development (NICHD) contributed 34 papers. Other major funders include the FWO (Belgium, 23), the National Science Foundation (NSF, USA, 19), and the European Union (16).

**Table 6:** Top 10 most productive funding agencies.

Rank	Funding agencies	Nations	Record count
1	United States Department of Health Human Services	USA	92
2	National Institutes of Health NIH USA	USA	87
3	NIH Eunice Kennedy Shriver National Institute of Child Health Human Development NICHD	USA	34
4	FWO	Belgium	23
5	National Science Foundation NSF	USA	19
6	European Union EU	EU	16
7	NIH National Institute of Neurological Disorders Stroke NINDS	USA	16
8	Conselho Nacional de Desenvolvimento Cientifico e Tecnologico CNPQ	Brazil	14
9	National Research Foundation of Korea	KOREA	13
10	Netherlands Organization for Scientific Research NWO	Netherlands	13

### 3.8 Dual-map overlay

**Figure 8:**
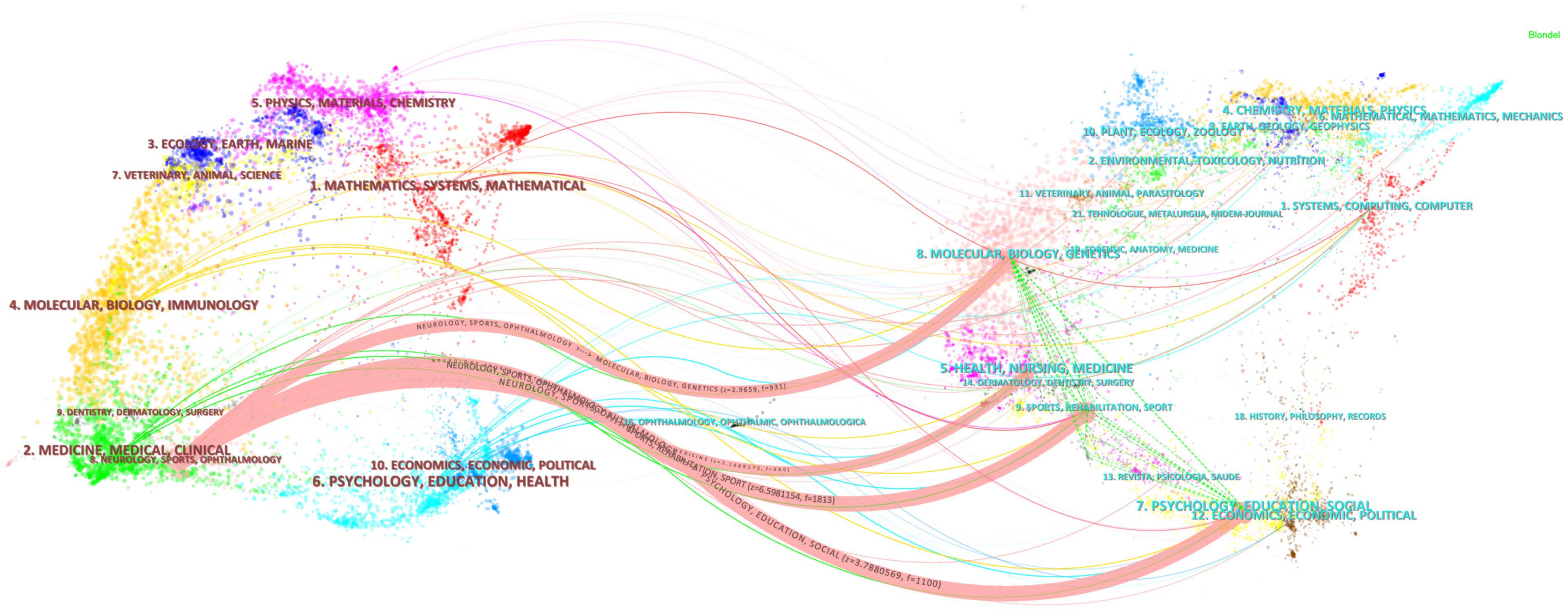
Dual-map overlay of journals. Citing journals are shown on the left, cited journals on the right; colored paths represent citation flows. The overlay reveals interdisciplinary knowledge transfer among neurology, rehabilitation, biomechanics, and engineering journals.

The dual-map overlay of journals reveals the cross-disciplinary knowledge flow of CP gait research, as shown in Figure 8. Citing journals are mainly concentrated in Neurology/Sports/Ophthalmology, Sports Rehabilitation, and Psychology/Education/Health. Cited journals, however, span a wide range of basic and applied disciplines: Molecular/Biology/Immunology, Physics/Materials/Chemistry, Environmental/Toxicology/Nutrition, and even Plant/Ecology/Zoology and Veterinary/Animal Science. This pattern confirms that CP gait analysis is not only a clinical field but also a strongly interdisciplinary domain that constantly absorbs techniques and concepts from basic life sciences and engineering. The dual-map overlay was generated using CiteSpace‘s default settings based on JCR categories, with citation links aggregated at the journal level.

### 3.9 Thematic evolution and research frontiers

**Figure 9:**
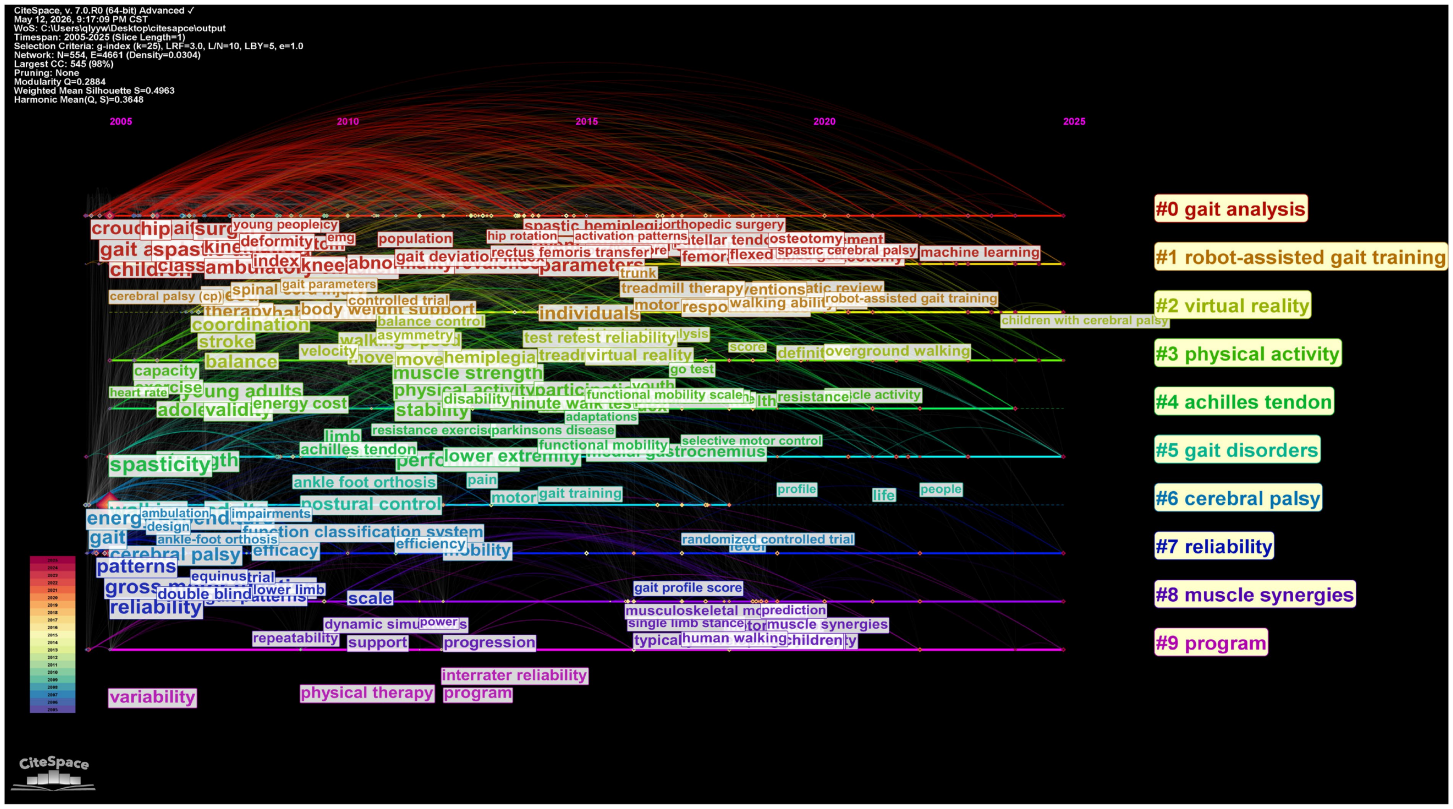
Timeline visualization of keyword clusters. Each horizontal line represents a cluster; nodes are keywords appearing in that year.

The timeline visualization of keywords (Figure 9) shows that clusters #0 (gait analysis) and #6 (cerebral palsy) have remained continuously active for the longest period, representing the persistent knowledge backbone of the field. Clusters related to “robot-assisted gait training” and “virtual reality” appear in more recent time windows, indicating a clear shift from traditional descriptive gait analysis toward technology-assisted rehabilitation. Recent burst keywords include “AI rehabilitation,” “wearable sensors,” and “deep learning” – all reflecting growing research interest in intelligent, digital, and precision approaches, although their clinical adoption remains limited.

### 3.10 Validation of bibliometric findings using Scopus

To assess the robustness of our WoSCC results, we performed a parallel bibliometric search in Scopus using the identical search strategy, time frame (2005–2025), and language restriction (English). Table 7 summarises the cross-database comparison of key indicators. Scopus retrieved 1820 records, ~30% more than WoSCC (1404), with 1,095 documents (78% of WoSCC records) overlapping. This indicates that while Scopus covers a broader journal spectrum, the core WoSCC-derived findings remain largely stable. Both databases consistently identified *Gait & Posture* as the most productive journal (207 articles in WoSCC vs. 260 in Scopus) and Vrije Universiteit Amsterdam as the leading institution. The top three productive countries were USA and the Netherlands in both, but China replaced Switzerland in Scopus, suggesting that WoSCC may under-index some Chinese journals. Keyword clustering showed perfect agreement on the dominant cluster “crouch gait / gait analysis,” confirming the thematic core. Emerging burst terms overlapped on “wearable sensors,” while Scopus additionally captured “digital biomarker,” reflecting its broader engineering coverage. The most highly cited article [Wren et al. (20)] ranked first in both databases, despite absolute citation counts differing (400 in WoSCC vs. 520 in Scopus). Finally, annual publication trends were almost perfectly correlated across databases (Spearman’s *ρ* = 0.98, *p* < 0.001). Collectively, this validation confirms that the principal conclusions of our WoSCC-based analysis—growth trajectory, leading journals/institutions, core themes, and emerging technologies—are robust and not artefacts of a single database. Minor discrepancies mainly arise from differential journal indexing policies and do not alter the original interpretation.

**Table 7:** WoSCC vs. Scopus validation: cross-database comparison of key bibliometric indicators.

Indicator	WoSCC	Scopus	Overlap/agreement	Interpretation
Total publications	1,404	1,820	1,095 (78%)	Scopus covers ~30% more records; core findings robust
Study period coverage	2005–2025	2005–2025	Identical	Consistent coverage; comparable baseline
Top 1 productive journal	*Gait & Posture* (207 articles)	*Gait & Posture* (260 articles)	Fully consistent	Flagship journal status confirmed across both databases
Top 3 productive countries	USA, Netherlands, Switzerland	USA, China, Netherlands	USA & Netherlands shared	China ranks higher in Scopus; WoS may under-index Chinese journals
Top institution	Vrije Universiteit Amsterdam	Vrije Universiteit Amsterdam	Fully consistent	Institutional leadership confirmed in both databases
Keyword cluster #1 label	Crouch gait / gait analysis	Crouch gait / gait analysis	Fully consistent	Core clinical themes stable across databases
Emerging keyword burst (2022–2025)	Wearable sensors, classification	Wearable sensors, digital biomarker, classification	Wearable sensors shared	Scopus captures additional engineering frontier terms
Top 1 highly cited article	Wren et al. (20) (400 citations)	Wren et al. (20) (520 citations)	Fully consistent	High-impact classics confirmed; citation counts differ by indexing
Unique articles (1 DB only)	309 WoSCC-only	725 Scopus-only	N/A	Unique records reflect different journal indexing policies
Annual trend correlation (Spearman)	*r* = 0.97 (*p* < 0.001)	*r* = 0.96 (*p* < 0.001)	rho = 0.98 (inter-database)	Publication growth trend highly consistent between databases

## 4 Discussion

This bibliometric study provides the first systematic mapping of CP gait research from 2005 to 2025. Over the 20-year period, annual output increased more than fourfold, confirming sustained research activity. The USA leads in absolute production, but European institutions—particularly Vrije Universiteit Amsterdam and KU Leuven—emerge as highly influential hubs, with above-average citation impact. *Gait & Posture* serves as the core specialty journal, while *Developmental Medicine and Child Neurology* achieves the highest per-article citations, reflecting the clinical relevance valued by the pediatric neurology community.

Keyword co-occurrence and clustering reveal that traditional instrumented gait analysis—three-dimensional motion capture force plates and surface electromyography—remains the dominant methodological core as evidenced by the persistent frequency of terms such as “reliability” “kinematics” and “spastic diplegia.” Alongside this established core keyword bursts since 2020 indicate rising research interest in “wearable sensors” “musculoskeletal modeling” and “artificial intelligence.” However their relatively lower overall frequencies and the predominance of small-scale proof-of-concept studies suggest that these technologies are not yet ready for routine clinical adoption—a distinction that is critical for clinicians and gait laboratory directors. The field is thus in a transitional research phase where novel engineering solutions attract growing attention but have not yet been integrated into standard care pathways

Several recent bibliometric studies have addressed CP-related topics, but none has specifically mapped instrumented gait analysis as a distinct domain (13–15). In contrast to these broader analyses, our study provides the first dedicated mapping that integrates knowledge structure, collaboration networks, and burst detection specifically for CP gait research. Beyond this thematic focus, our cross-validation between VOSviewer and CiteSpace—a methodological step not consistently applied in previous CP bibliometric studies—enhances the reliability of our network visualizations and allows us to identify evidence gaps (e.g., the scarcity of intervention-focused publications) that are often obscured in broader analyses.

Beyond these patterns, the publication record reveals a notable thematic imbalance: the majority of retrieved original articles (>70%) are cross-sectional or descriptive, focusing on characterizing gait deviations or establishing measurement reliability. In contrast, intervention studies that directly evaluate the effect of gait-analysis-guided treatments, such as pre- vs. post-operative outcomes following orthopaedic surgery, or comparative effectiveness of botulinum toxin injection protocols informed by three-dimensional gait analysis, remain substantially underrepresented. A recent systematic review on femoral derotation osteotomy in CP noted that while gait analysis is widely used in preoperative planning, only a minority of studies report prospective, standardized protocols linking kinematic data to surgical decision algorithms (27). Similarly, clinical practice guidelines for CP gait management emphasize the need for more rigorous evidence on how instrumented gait analysis changes therapeutic choices and ultimately improves patient-centered outcomes (28). We therefore interpret this pattern as a publication-level signal that intervention-focused and implementation-oriented research remains an underdeveloped area, warranting prioritized funding and multi-center collaboration.

Despite this thematic imbalance, the mapping of collaborative structures and publication outlets offers immediate practical value for researchers and clinicians. These resources can be strategically leveraged to address the very gaps identified above—for instance, by facilitating targeted collaborations between established hubs and emerging laboratories, or by guiding evidence-based curriculum design. The identification of core journals (*Gait & Posture*, *Developmental Medicine and Child Neurology*, *Clinical Biomechanics*), leading institutions (Vrije Universiteit Amsterdam, KU Leuven, Gillette Children‘s Specialty Healthcare), and productive authors (Desloovere K, Buizer AI, Lerner ZF) offers practical references for clinicians, laboratory directors, and rehabilitation educators seeking to establish reading lists, identify collaboration partners, or benchmark performance. The dense collaboration networks among Western European and North American institutions highlight the value of cross-center partnerships; newly established gait laboratories, particularly in underrepresented regions, could benefit from twinning programs with these hubs. Additionally, the growing application of bibliometric methods suggests that training curricula for clinical gait analysts and physical therapists could include basic science mapping skills—such as interpreting keyword burst detection and collaboration networks—to help practitioners efficiently navigate the expanding literature and identify emerging evidence before it becomes mainstream.

Several limitations should be acknowledged. First, data were sourced exclusively from the Web of Science Core Collection, which may underrepresent regional journals and non-English publications, potentially skewing the global representation of research activity. Second, our broad search strategy (“gait” and “walking”) maximized sensitivity but may have included studies without instrumented biomechanical assessment; the results should therefore be interpreted as reflecting gait-related CP research broadly, rather than exclusively instrumented gait analysis. Third, bibliometric indicators reflect publication and citation patterns, not the clinical validity or methodological quality of individual studies; high citation counts do not equate to clinical utility. Fourth, the cross-sectional nature of bibliometric analysis captures historical patterns and cannot predict future directions with certainty. Fifth, while we followed PRISMA for search and selection transparency, this study did not perform a qualitative risk-of-bias assessment, as that is beyond the scope of bibliometric mapping.

Building on these findings, we propose two priority directions. First, multi-center, large-sample gait data sharing initiatives should be expanded. Most existing studies are single-center with modest sample sizes (median N < 30), limiting generalizability. International consortia pooling instrumented gait data would enable more robust subgroup classification, powered intervention studies, and validation of AI-based prediction models. Second, real-world evidence and clinical decision support systems should be developed. Rather than reporting only group-level statistics, future work should focus on integrating gait analysis outputs into electronic health records and generating patient-specific recommendations (e.g., predicting crouch gait progression risk or optimal surgical timing). Embedding gait analysis within learning health systems represents a concrete pathway to bridge the current translation gap.

In summary, this bibliometric analysis reveals that CP gait research has matured from a descriptive, laboratory-based activity to a field increasingly engaged with technological innovation. However, the gap between research output and clinical implementation persists, and emerging technologies, while promising, require rigorous validation before broad adoption. The collaborative networks and thematic hotspots identified here provide a roadmap for strategic investment and interdisciplinary partnership in the coming decade.

## 5 Conclusion

This bibliometric analysis confirms steady growth in cerebral palsy gait research (26–112 annual publications, 2005–2025) and a transition toward technology-assisted rehabilitation. Yet a critical clinical gap remains: descriptive studies dominate (>70%), while evidence linking instrumented gait analysis to patient-centered outcomes is lacking. For clinicians, wearable sensors, AI, and musculoskeletal modeling are emerging but not yet ready for routine adoption. Methodologically, the combined use of VOSviewer and CiteSpace with cross-validation of network structures represents an innovative approach that enhances the reliability of our mapping. Future efforts should prioritize multi-center validation and clinical decision support tools that translate gait metrics into individualized treatment plans.

## Data Availability

The original contributions presented in the study are included in the article/Supplementary material, further inquiries can be directed to the corresponding author.
