# Supplementary figures and images for: Gait analysis in cerebral palsy (2005–2025): a bibliometric mapping of research trends, collaboration networks, and emerging technologies
Source: Front Neurol. 2026 Jul 7;17:1899328. doi: 10.3389/fneur.2026.1899328 (PMC13384833; doi:10.3389/fneur.2026.1899328)

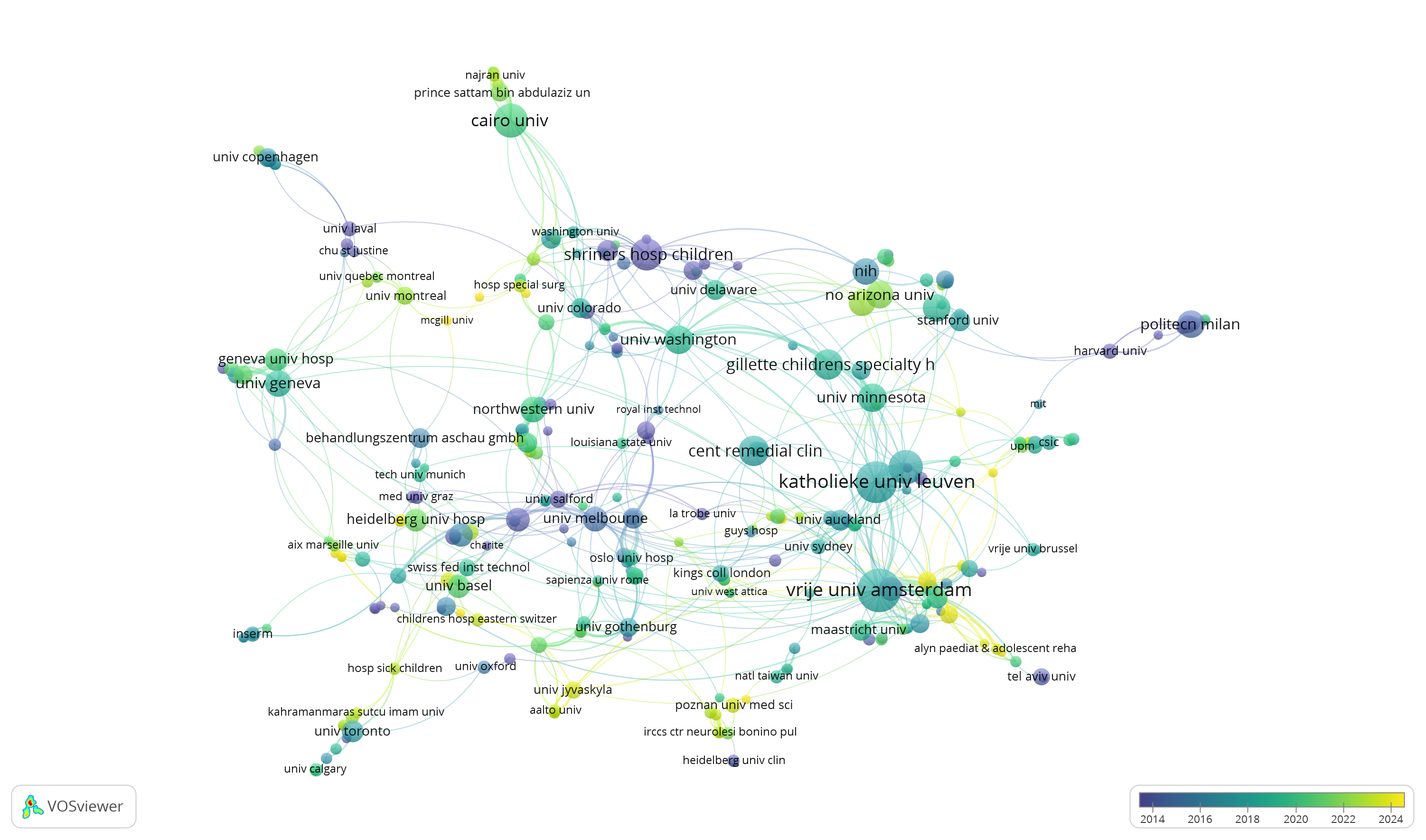

Supplement: Supplementary file 1 [file Image_1.TIFF]
