# Supplementary material for: Gait analysis in cerebral palsy (2005–2025): a bibliometric mapping of research trends, collaboration networks, and emerging technologies
Source: Front Neurol. 2026 Jul 7;17:1899328. doi: 10.3389/fneur.2026.1899328 (PMC13384833; doi:10.3389/fneur.2026.1899328)
Supplement: Supplementary file 2 [file Table_1.DOCX]

**Supplementary Table S1. PRISMA 2020 Checklist**

| **Section and Topic** | **Item #** | **Checklist item** | **Location where item is reported** |
| --- | --- | --- | --- |
| **TITLE** | | | |
| Title | 1 | Identify the report as a systematic review. | Title page (explicitly states "systematic review" and "bibliometric analysis") |
| **ABSTRACT** | | | |
| Abstract | 2 | See PRISMA 2020 for Abstracts checklist. | Abstract (structured: Background, Methods, Results, Conclusion) |
| **INTRODUCTION** | | | |
| Rationale | 3 | Describe the rationale for the review in the context of existing knowledge. | Introduction, paragraphs 1-4 |
| Objectives | 4 | Provide an explicit statement of the objective(s) or question(s) the review addresses. | Introduction, final paragraph (four specific aims) |
| **METHODS** | | | |
| Eligibility criteria | 5 | Specify the inclusion and exclusion criteria for the review. | Section 2.2 (original articles/reviews; English; 2005-2025; topic focus) |
| Information sources | 6 | Specify all databases, registers, and other sources searched. | Section 2.1 (Web of Science Core Collection only) |
| Search strategy | 7 | Present the full search strategy for at least one database. | Section 2.1 (search syntax: TS = ("cerebral palsy" OR "CP") AND TS = ("gait" OR "walking" OR "gait analysis")) |
| Selection process | 8 | Specify the methods used to decide whether a study met the inclusion criteria. | Section 2.2 (title/abstract screening by two authors; disagreements resolved by discussion) |
| Data collection process | 9 | Describe the methods used to collect data from reports. | Section 2.2 (data exported as full record and cited references; deduplication; normalization) |
| Data items | 10a | List and define all outcomes for which data were sought. | Not applicable (bibliometric analysis – outcomes are publication counts, citations, collaboration networks, etc.) |
|  | 10b | List and define all other variables for which data were sought (e.g., funding, study characteristics). | Section 2.3 (authors, institutions, countries, journals, keywords, co-citation, funding agencies) |
| Study risk of bias assessment | 11 | Specify the methods used to assess risk of bias in the included studies. | Not applicable – this is a bibliometric review, not a meta-analysis of intervention effects. However, methodological limitations are discussed in Discussion (limitations paragraph) |
| Effect measures | 12 | Specify for each outcome the effect measure(s) (e.g., risk ratio, mean difference). | Not applicable |
| Synthesis methods | 13a | Describe the processes used to decide which studies were eligible for each synthesis. | Section 2.2 and 2.3 (all retrieved records meeting inclusion criteria were analysed) |
| Synthesis methods | 13b | Describe the methods used to prepare and summarise data for synthesis. | Section 2.3 (VOSviewer, CiteSpace; co-authorship, co-occurrence, clustering, burst detection) |
|  | 13c | Describe the methods used to tabulate or visually display results. | Section 3 (figures and tables as indicated) |
|  | 13d | Describe the methods used to synthesise results. | Section 2.3-2.4 (linear regression for time trends, network analysis parameters) |
|  | 13e | If meta-analysis was performed, describe the methods. | Not applicable |
|  | 13f | Describe the methods used to explore possible causes of heterogeneity among study results. | Not applicable |
|  | 13g | Describe any sensitivity analyses used to assess robustness of the synthesised results. | Not performed – but the choice of g-index, k-core, and Pathfinder pruning is described in Section 2.3 |
| Reporting bias assessment | 14 | Describe the methods used to assess risk of bias due to missing results in the synthesis. | Not applicable (bibliometric data are complete from WoSCC for the defined period) |
| Certainty assessment | 15 | Describe the methods used to assess certainty (or confidence) in the body of evidence. | Not applicable |
| **RESULTS** | | | |
| Study selection | 16a | Describe the results of the search and selection process. | Section 3.1 and Figure 1 (PRISMA flow diagram: 1404 articles included) |
|  | 16b | Cite the PRISMA flow diagram. | Figure 1 (PRISMA 2020 flow diagram) |
| Study characteristics | 17 | Cite each included study and present its characteristics. | Not applicable (aggregated bibliometric data; all 1404 studies are listed in WoSCC, but individual characteristics are not reported) |
| Risk of bias in studies | 18 | Present assessments of risk of bias for each included study. | Not applicable |
| Results of individual studies | 19 | For all outcomes, present, for each study, summary statistics. | Not applicable |
| Results of syntheses | 20a | For each synthesis, summarise the characteristics and results. | Section 3.2-3.8 (publication trends, country/institution/author/journal rankings, collaboration networks, keyword clusters, bursts, co-citation, funding, dual-map) |
| Results of syntheses | 20b | If meta-analysis was performed, present summary estimates. | Not applicable |
|  | 20c | Present results of all investigations of possible causes of heterogeneity. | Not applicable |
|  | 20d | Present results of all sensitivity analyses. | Not applicable |
| Reporting biases | 21 | Present assessments of risk of bias due to missing results. | Not applicable |
| Certainty of evidence | 22 | Present assessments of certainty (or confidence) in the body of evidence. | Not applicable |
| **DISCUSSION** | | | |
| Discussion | 23a | Provide a general interpretation of the results. | Discussion, paragraphs 1-2 |
| Discussion | 23b | Discuss any limitations of the evidence. | Discussion, limitations paragraph (WoSCC only, citation bias, cross-sectional nature, no bias assessment) |
|  | 23c | Discuss any limitations of the review methods. | Discussion, limitations paragraph (as above) |
|  | 23d | Discuss implications of the results for practice, policy, and future research. | Discussion, paragraphs 3-5 (practical insights for clinicians, future priorities: multi-center data sharing, real-world evidence) |
| **OTHER INFORMATION** | | | |
| Registration and protocol | 24a | Provide registration information for the review, including register name and registration number. | Section 2.1 (preregistered on OSF; DOI: 10.17605/OSF.IO/CT27P) |
| Registration and protocol | 24b | Indicate where the review protocol can be accessed, or state that a protocol was not prepared. | Section 2.1 (OSF link provided) |
|  | 24c | Describe and explain any amendments to the protocol. | No amendments |
| Support | 25 | Describe sources of financial or non-financial support for the review. | Conflict of Interest statement (none declared) and acknowledgement section if any – note: none explicitly stated, but "the authors declare no conflict of interest" |
| Competing interests | 26 | Declare any competing interests of review authors. | Conflict of Interest statement |
| Availability of data, code, and other materials | 27 | Report which of the following are publicly available and where they can be found: data, code, other materials. | Data Availability Statement (raw data from WoSCC available upon request; search syntax and R script in Supplementary Material; protocol on OSF) |
