# Supplementary material for: Gait analysis in cerebral palsy (2005–2025): a bibliometric mapping of research trends, collaboration networks, and emerging technologies
Source: Front Neurol. 2026 Jul 7;17:1899328. doi: 10.3389/fneur.2026.1899328 (PMC13384833; doi:10.3389/fneur.2026.1899328)
Supplement: Supplementary file 3 [file Table_2.doc]

**Detailed Parameter Settings for Bibliometric Analysis**

**1. CiteSpace Parameter Settings**

**CiteSpace** (version 7.0 R0) was configured with the following parameters for network construction and visualization:

**Time slicing**: 2005–2025, with 1‑year slices (consistent with the study period).

**Node types**: Author, institution, country, keyword, cited reference, cited journal (analyzed separately depending on the network type).

**Selection criteria**: The g‑index (k = 15) was applied for each annual slice. This threshold was selected based on a pilot analysis to balance the inclusion of emerging, low‑frequency terms against the generation of spurious clusters.

**Pruning**: The Pathfinder algorithm was used to prune the network, reducing visual clutter while preserving the backbone structure of co‑occurrence and co‑citation links.

**Clustering**: Keyword clusters were labelled using the log‑likelihood ratio (LLR) algorithm, which has been shown to produce more meaningful cluster labels compared to alternative methods such as Latent Semantic Indexing (LSI) or Mutual Information (MI).

**Cluster evaluation**: The final clustering solution was selected after several iterative calibrations to maximize both:

**Modularity** (Q): A measure of network partitioning quality, with values > 0.3 indicating significant community structure.

**Mean silhouette** (S): A measure of cluster coherence, with values > 0.5 indicating well‑defined clusters.

**Burst detection**: Keywords with a sudden increase in frequency over time were identified using CiteSpace’s burst detection algorithm, with a minimum burst duration of 1 year.

Dual‑map overlay: Generated using CiteSpace’s built‑in journal mapping based on JCR categories, with citation links aggregated at the journal level .

**2. VOSviewer Parameter Settings**

**VOSviewer** (version 1.6.20) was employed to complement and validate the CiteSpace networks with the following settings:

**Counting method**: Fractional counting was used for co‑authorship and co‑occurrence analyses to reduce the influence of multi‑authored or multi‑affiliated records.

Normalization: Association strength normalization was applied to construct the similarity matrices.

**Network types**:

Co‑authorship networks (author, institution, country)

Keyword co‑occurrence network

Journal bibliographic coupling network

Minimum cluster size: Default setting (minimum of 3 items per cluster) was applied for keyword clustering.

**3. Analytical Approach for Publication Trends**

Annual publication trends were analyzed using linear regression in R (version 4.3.0, R Core Team, 2023). The Durbin‑Watson statistic was calculated to test for autocorrelation in the residuals. The significance level for regression coefficients was set at p < 0.05.

**4. Scopus Validation Parameters**

To validate the robustness of the Web of Science Core Collection findings, a parallel search was performed in **Scopus** (Elsevier) using:

**Search syntax**: TITLE-ABS-KEY ("cerebral palsy" OR "cp") AND TITLE-ABS-KEY (gait OR walking OR "gait analysis")

**Time frame**: 2005–2025 (identical to WoSCC)

**Document types**: Articles and reviews (identical to WoSCC)

**Language**: English (identical to WoSCC)

**Overlap quantification**: DOI‑based matching as the primary key, with title–first‑author–year fuzzy matching for records without a DOI.
